# Reinforcing effects of methamphetamine in an animal model of Attention-Deficit/Hyperactivity Disorder-the Spontaneously Hypertensive Rat

**DOI:** 10.1186/1744-9081-6-72

**Published:** 2010-12-09

**Authors:** Ike dela Peña, Hyung Seok Ahn, Ji Young Choi, Chan Young Shin, Jong Hoon Ryu, Jae Hoon Cheong

**Affiliations:** 1 Uimyung Research Institute for Neuroscience, Sahmyook University, 26-21 Kongkreung-dong, Nowon-gu Seoul 139-742, Korea; 2 Center for Geriatric Neuroscience Research, Institute of Biomedical Science and Technology, Konkuk University, Seoul, 143-701 Korea; 3 Department of Oriental Pharmaceutical Science, Kyunghee University, Dongdaemun-gu, Hoegidong, Seoul 130-701, Korea

## Abstract

Substrains of the Spontaneously Hypertensive rat (SHR), a putative animal model of Attention-Deficit/Hyperactivity Disorder (ADHD), have demonstrated increased sensitivity to many drugs of abuse, including psychostimulants. Therefore, it was suggested that studies in SHR may help elucidate ADHD and comorbidity with substance use disorder (SUD). However, the drug intake profile of the SHR in the most relevant animal model of drug addiction, the self-administration (SA) test, and its response on the conditioned place preference (CPP) paradigm are not yet determined. In the present study, we employed SA and CPP tests to investigate the reinforcing effects of the psychostimulant methamphetamine in an SHR substrain obtained from Charles River, Japan (SHR/NCrlCrlj). Concurrent tests were also performed in Wistar rats, the strain representing "normal" heterogeneous population. To address if the presence of ADHD behaviors further increases sensitivity to the rewarding effect of methamphetamine during adolescence, a critical period for the onset of drug abuse, CPP tests were especially conducted in adolescent Wistar and SHR/NCrlCrlj. We found that the SHR/NCrlCrlj also acquired methamphetamine SA and CPP, indicating reinforcing effects of methamphetamine in this ADHD animal model. However, we did not observe increased responsiveness of the SHR/NCrlCrlj to methamphetamine in both SA and CPP assays. This indicates that the reinforcing effects of methamphetamine may be similar in strains and that the SHR/NCrlCrlj may not adequately model ADHD and increased sensitivity to methamphetamine.

## Findings

Attention-Deficit/Hyperactivity Disorder (ADHD) is a complex neurodevelopmental disorder characterized by the core symptoms such as hyperactivity, inattention and impulsivity [1,2]. It is the most commonly diagnosed disorder of childhood [3] and also present in about 4%-9% of youths [4,5] and 4% of adults [6]. ADHD is comorbid with substance use disorder (SUD) [7,8] and epidemiological data not only affirm the ADHD-SUD link but also indicate greater risk for earlier onset of substance abuse among ADHD individuals [4,9-11]. The exact etiology, however, cannot be determined for in some respects, there is a lack of appropriate animal models [12].

The Spontaneously Hypertensive rat (SHR), bred from the normotensive Wistar Kyoto (WKY) rat strain, is the most validated animal model of ADHD [13]. A number of SHR substrains exist [14] and Sagvolden *et al *[15] asserted that the SHR obtained from Charles River, Germany (SHR/NCrl), with the WKY bred from Harlan, UK (WKY/NHsd) as the reference strain, most excellently represents ADHD. Nevertheless, earlier studies have found increased reactivity of some SHR substrains (e.g. SHR/NTac, SHR/NCrl) to stimulants, opioids, alcohol and other addictive drugs in comparison with other rat strains [see [12] for review], indicating that the SHR, in general, may be used to investigate the relationship between ADHD and drug addiction. However, the behavioral profiles of the SHR in the most relevant drug addiction assay, the self-administration (SA) paradigm [12] and in another animal model of addiction, the conditioned place preference (CPP) protocol [16] are not completely known.

In this study, we conducted SA and CPP tests in an SHR substrain obtained from Charles River, Japan (SHR/NCrlCrlj) (via Orient Bio., Korea), to investigate if it shows stable responding for the stimulant methamphetamine. Its response was compared with the Wistar rat, strain representing the "normal" heterogeneous population [17-19]. Adolescence is a risk factor for addiction [20] and it is known that children with ADHD are more vulnerable to use illicit drugs than children without it [21,22]. We would like to know if we can model this in our study, thus CPP tests were conducted in adolescent Wistar and SHR/NCrlCrlj.

All experiments complied with the Principles of Laboratory Animal Care (NIH) and the Animal Care and Use Guidelines of Sahmyook University, Korea. We used adolescent (PND 20-47) and adult (PND 90-110) male outbred Wistar and inbred SHR/NCrlCrlj. They were housed collectively (8 rats/cage in CPP experiments) or individually (SA experiments), in a temperature (22 ± 2°C) and humidity- (55 ± 5%) controlled animal room with a 12 hr/12 hr light/dark (6 AM-6 PM) cycle. They had free food and water access except during tests and on initial lever-training sessions (SA test). Methamphetamine hydrochloride, procured from Korea Food and Drug Administration, was dissolved in saline for use in all experiments.

Self-administration tests were carried out in standard operant chambers (Coulbourn Instruments, Allentown, PA, USA) and the methods employed were as outlined previously [23]. Briefly, after rats acquired stable lever responding, they were implanted with silastic catheters in the right jugular vein. SA tests commenced after they have recovered from surgery. Two levers were present during SA tests and response on the left lever (active lever, FR1) switched on the infusion pump for 10 s, delivering 0.1 ml of 0.25 mg methamphetamine, and also the stimulus light above it (which was lit for 10 s and 20 s more after the end of the infusion). A time-out period was indicated and no priming injections were given at any time. Rats were only allowed up to 30 infusions although lever responses were recorded until the end of the 2-hour SA. Catheter patency was ensured as detailed [23].

Conditioned place preference tests were conducted in two-compartment polyvinylchloride boxes having distinct visual and tactile cues. The methods were patterned after previous reports [23,24] with some modifications. After determining the rats' initially-preferred compartment, approximately half of the rats per group were assigned to the black compartment as the drug-paired side, while the other half to the other [24]. If their staying time was less than 200 s, they were excluded from further testing. Conditioning phase followed where animals were paired with methamphetamine (1.25 or 5 mg/kg) or saline in their non-preferred compartment. The rest of the procedures were performed as described [23]. Post- and preconditioning staying times were determined using automated systems (Ethovision Noldus IT b.v., Netherlands).

Two-way ANOVA was used to determine the effects of strains or days or interaction between these factors in SA tests (active *vs *inactive lever presses, and total number of methamphetamine infusions) and also in CPP tests (strains or treatment effects and interaction between them). Student's *t*-test was used for further analysis. The significance level was set to p < 0.05.

**Figure 1:**
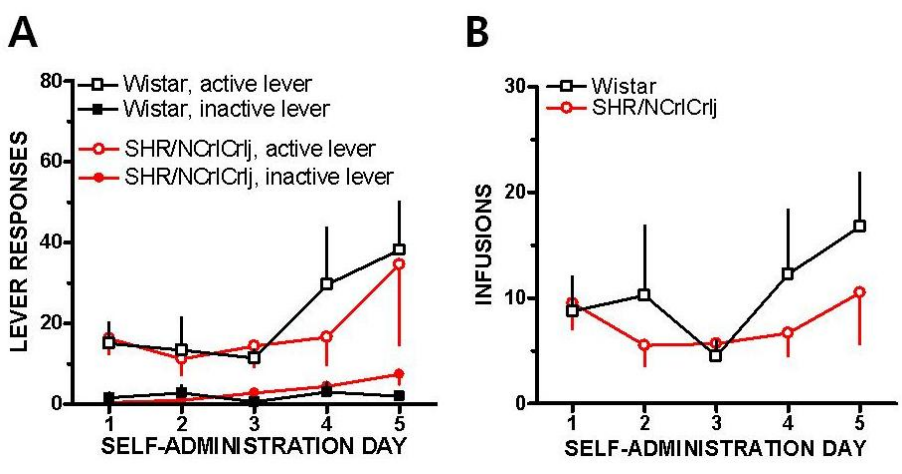
**Acquisition of methamphetamine self-administration (SA)**. (A) Mean total number of lever responses of Wistar rats and SHR/NCrlCrlj during the 2-hour methamphetamine SA. Open symbols indicate lever responses on the active (reinforced) lever and filled symbols show responses for the inactive (non-reinforced) lever. (B) Mean infusions obtained by Wistar and SHR/NCrlCrlj during the 2-hour methamphetamine self-administration. Empty squares are responses of Wistar rats and circles are for SHR/NCrlCrlj. Data are presented as means ± SEM (*n *= 6).

Figure 1A shows that both strains acquired methamphetamine SA. Responses for the active *vs *inactive lever differed significantly in Wistar (F (1,50) = 59.92, p < 0.001) and in SHR/NCrlCrlj (F (1,50) = 42.26, p < 0.001). However, two-way ANOVA only revealed significant effect of days (F (4,50) = 5.69, p < 0.001), but without any strain × days interaction. In Figure 1B, the number of methamphetamine infusions earned by SHR/NCrlCrlj and Wistar rats during the SA tests is shown. Significant days (F (4,50) = 7.01, p < 0.001) but not strain effects was noted, and there was no strain x days interaction.

**Figure 2:**
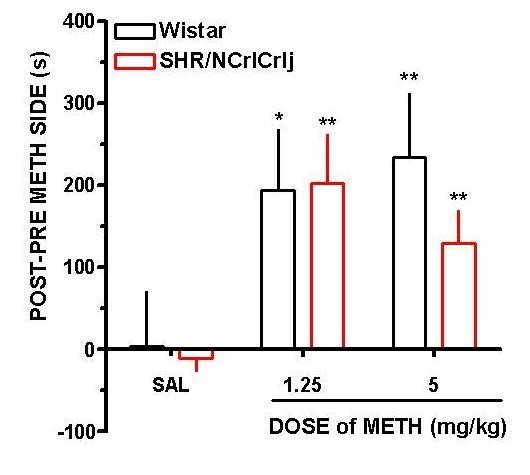
**Rewarding effects of methamphetamine (METH) in Wistar and SHR/NCrlCrlj**. (A) Place preference data (mean ± SEM) expressed as the difference in the amount of time spent in the METH-paired compartment during the post- and preconditioning days. Data are expressed as mean ± SEM. *p < 0.05, **p < 0.01 when comparing saline (SAL) *vs *METH-conditioned animals (*n *= 8).

Figure 2 illustrates that CPP was expressed by both strains in response to methamphetamine conditioning. There was a remarkable effect of treatment (F (2,42) = 6.46, p < 0.05), but no strain effects, and strain × treatment interaction. In Wistar rats, there was CPP to 1.25 mg/kg (t (14) = 1.81, p < 0.05) and 5 mg/kg (t (14) = 2.17, p < 0.05) methamphetamine. CPP was also demonstrated by SHR/NCrlCrlj conditioned with 1.25 (t (14) = 3.33, p < 0.01) and 5 mg/kg (t (14) = 2.96, p < 0.01) methamphetamine.

This study showed that the SHR/NCrlCrlj, alike the Wistar rat, readily acquired methamphetamine self-administration. Adolescent SHR/NCrlCrlj also demonstrated conditioned place preference to methamphetamine although in both assays differential strain response was not observed. It indicates that the reinforcing/rewarding properties of methamphetamine are similar in both strains and more importantly, that the SHR/NCrlCrlj may not be more sensitive to methamphetamine reward/reinforcement. The results herein do not conform to previous investigations. Initially, it has been predicted that the SHR would show increased behavioral response to addictive drugs not only for their ADHD-like behaviors, but also as it manifests high levels of impulsivity, novelty-seeking behaviors [25] and defective/deficient reinforcement processes [26]. These behaviors predispose greater vulnerability to drug abuse as known in human studies [27,28]. Secondly, our results do not coincide with previous studies (which used locomotor activity as an index to the reinforcing effects of drugs) which demonstrated increased behavioral response of an SHR substrain (SHR/NTac) to amphetamine, methylphenidate and the selective D1 receptor agonist SKF-81297, as compared to Wistar Kyoto rats (WKY/NTac) [29-32]. However, one study has found similarity of the effects of amphetamine or GBR-12909, a dopamine reuptake inhibitor, in another SHR substrain (SHR/Cbp) and in WKY/Cbp [33]. The present inconsistency could be due to the following factors: (1) difference in procedures (*i.e*. locomotor activity and SA tests measure different aspects of reinforcement mechanism) and (2) difference in SHR substrains used (*i.e*. the SHR substrain used herein may deviate phenotypically or genetically from SHR/NCrl bred in the USA or elsewhere) [15]. Nevertheless, it has to be considered that stimulants act differently in exerting their reinforcing effects [34]. This factor may also have influenced the present results. At any rate, additional investigations should be performed to resolve this discrepancy.

Our CPP results do not model increased vulnerability of ADHD children to drugs of abuse in comparison with "normal" children [21,22]. However, our data may represent a previous study which showed no difference in the rates of drug abuse or dependence to individual substances in both ADHD and non-ADHD controls [35]. Of interest is that our CPP data agree with those obtained in SA test. This is notable considering that CPP and SA are believed to be dissimilar forms of drug reward [36-38].

In summary, we have shown that the SHR/NCrlCrlj also shows stable responding and CPP to methamphetamine. However, we have not observed increased sensitivity of this substrain to methamphetamine reward/reinforcement relative to Wistar rats, showing that it may not fully represent ADHD and increased vulnerability to methamphetamine. Concerning methamphetamine-induced CPP in SHR/NCrlCrlj, it cannot be attributed to an anxiolytic effect of repeated methamphetamine treatment [see [39]], as drug-induced anxiolysis (which may influence place preference) is not always apparent with drug-induced decreases in central serotonin [40]. It could not be due to "response to novelty" [36] as the CPP procedure was modified to minimize the effect of this confound. In future studies, it would be worthwhile to compare the behavioral responses of the SHR/NCrlCrlj or SHR/NCrl (of Charles River, Germany) with its most appropriate control strain, the WKY/NHsd, in both SA and CPP assays. Such work may not only determine the reliability of the present conclusion, but may also give clues on the contribution of neurobiological differences (seen in ADHD and non-ADHD individuals) in response to the reinforcing effects of drugs of abuse.

## Competing interests

The authors declare that they have no competing interests.

## Authors' contributions

ID and JHC conceived and designed the study. ID, HSA and JYC performed the SA and CPP experiments. ID performed the statistical analysis while SCY, JHR JHC provided insights on interpreting the results. All authors participated in drafting and in writing the final version of the manuscript.
